# Women Pharmacists and Hospital Dispensing

**Published:** 1912-08-31

**Authors:** Geo. W. Udale

**Affiliations:** Pharmaceutical Chemist. Ellastone, Harlington, Middlesex


### The Editor's Letter-Box.

### Women Pharmacists and Hospital Dispensing.

*To the Editor of* The Hospital.

Sir,—Adverting to your leaderettes anent the above, in your issue of the 24th inst., it might be of interest to your readers to learn that the 1911 Register of Pharmaceutical Chemists and Chemists and Druggists contained the names of 282 women pharmacists. Thirty-six women were registered as pharmaceutical chemists and 246 as chemists and druggists. I will not vouch for the strict accuracy of these figures, but they may be accepted as being somewhere very near the mark. It will thus be noted that women pharmacists are not numerically strong, but I see no cogent reason why a woman pharmacist should not, in the near future, aspire to the honourable position of a councillor, or even the presidency, of the Pharmaceutical Society of Great Britain. I have seen so many cases within my own small "ken" in which the situation has been so frequently saved by woman, that I consider the fullest honours should be open to woman in every sphere of life; and, so long as women fight for equal rights, especially as regards the price of labour, alongside of men, so long will they receive my support and vote.

The remarks of Mr. Saville Peck concerning the hospital pharmacy or dispensary as not being a suitable training-ground for pharmaceutical pupils are unworthy of so erudite a gentleman. It is obvious to anyone in a position to know that the only missing link in this direction is the art of salesmanship; in every other department, professional or business, the institutional pharmacy presents facilities which, to say the least, are not outrivalled by 10 per cent. of the retail pharmacies of the country.—Yours faithfully,

Geo. W. Udale, M.P.S.,
Pharmaceutical Chemist.

Ellastone, Harlington, Middlesex, August 24.

